# Pyloric Gland Metaplasia of the Ileocecal Valve: Clinicopathologic Correlates of Inflammatory Bowel Disease

**DOI:** 10.7759/cureus.1817

**Published:** 2017-11-03

**Authors:** Daryl Ramai, Kinesh Changela, Madhavi Reddy

**Affiliations:** 1 Division of Gastroenterology and Hepatology, Academic Affiliate of the Icahn School of Medicine, Clinical Affiliate of the Mount Sinai Hospital

**Keywords:** crohn’s disease, pyloric metaplasia, ileocecal valve, colonoscopy, inflammatory bowel disease

## Abstract

Pyloric gland metaplasia of the ileocecal valve, in the setting of Crohn’s disease, is an unusual clinical entity. Though its etiology and pathogenesis remains unclear, metaplastic changes have been associated with chronic inflammation and inflammatory bowel disease. Herein, we report a case of a 23-year-old male who presented for surveillance colonoscopy after being diagnosed with Crohn's disease four years ago. Diagnostic colonoscopy revealed stenosis of the ileocecal valve as well as a 5 mm polypoid circumferential non-obstructing lesion. Excisional biopsy followed by histopathology revealed pyloric metaplasia and non-necrotizing epithelioid cell granuloma. We discuss the clinical significance of pyloric gland metaplasia of the ileocecal valve in the context of inflammatory bowel disease.

## Introduction

The ileocecal valve is a sphincter that separates the small intestine from the large intestine. Its function includes limiting the reflux of colonic contents into the ileum and regulating small bowel contents that move to the large bowel. During a colonoscopy, the ileocecal valve serves as a landmark for colonoscopy completion. The histology of the ileocecal valve shows a change from the villous mucosa of the ileum to a more colonic mucosa, along with thickening of the muscularis mucosa. Thickening of the muscularis externa is also noted at the valve, in addition to variable amounts of lymphatic tissue. Herein, we present a case of pyloric gland metaplasia of the ileocecal valve in a patient diagnosed with Crohn’s disease. We present our findings following colonoscopy as well as histopathology which demonstrate the presence of mucin-secreting cells of the gastric pylorus, suggestive of pyloric metaplasia.

## Case presentation

**Figure 1:**
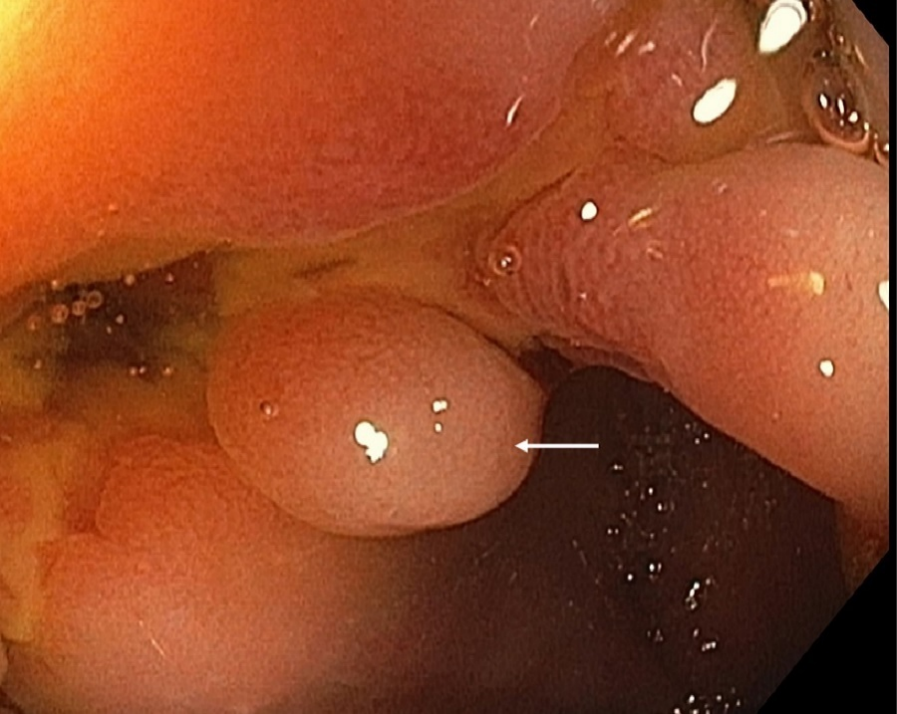
Endoscopic view of 5 mm polypoid lesion (arrow) at the ileocecal valve.

**Figure 2:**
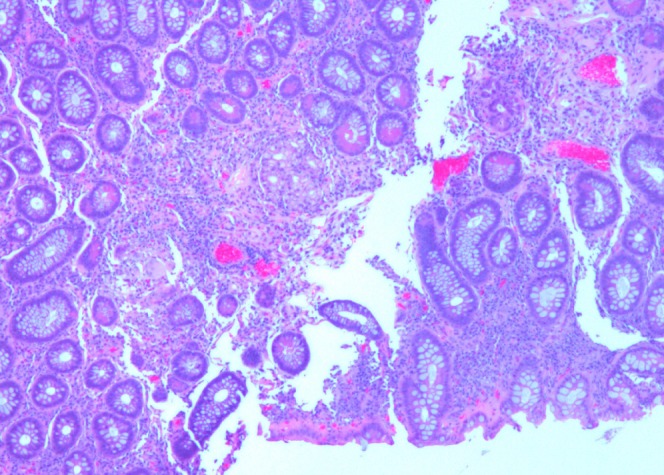
Ileocecal valve biopsy shows aberrant pyloric glands with a foamy appearance (hematoxylin and eosin stained, x40).

A 23-year-old male who was diagnosed with Crohn’s disease four years ago presented for surveillance colonoscopy. The patient was asymptomatic at the time of colonoscopy. The patient was a non-smoker and non-drinker, and review of systems was negative for any weight loss. Physical examination revealed mild diffuse lower abdominal tenderness. Vital signs were stable, and laboratory results were within normal limits. The patient underwent a colonoscopy which revealed a 5 mm polypoid circumferential non-obstructing lesion seen at the ileocecal valve (Figure 1). The ileocecal valve appeared ulcerated and stenotic. Biopsy of the lesion followed by histopathology revealed mildly active chronic colitis, pyloric metaplasia, and non-necrotizing epithelioid cell granuloma (Figure 2). 

## Discussion

Aberrant gastric glands are gastric glands found in organs other than the stomach. This rare condition has been classified into two categories: gastric heterotopia and gastric metaplasia. The former is a congenital type, while the latter is an acquired form. Furthermore, it is well known that gastric glands found in Meckel's diverticulum and gastric inlet patch indicate heterotopia. Though found much less frequently, intestinal duplication is another instance of gastric heterotopia [1].

As detected in our patient, the resemblance of cells to the mucin cells of the gastric pylorus forms the basis of pyloric metaplasia [2]. Metaplasia represents a plasticity of cellular architecture in which one cell type is replaced by another as an adaptive mechanism to withstand environmental stressors. Pyloric metaplasia is also referred to as pseudopyloric gland metaplasia or mucous gland metaplasia which reflects chronic mucosal inflammation [3]. This is associated with mucosal damage in cases of trauma, prolapse, and non-steroidal anti-inflammatory drug-induced injury [3-4].

Pyloric metaplasia is typically observed at the site of the terminal ileum. This feature is seen in 2-27% of ileal biopsies in patients with chronically active Crohn’s disease or patients with ileal pouch-anal anastomoses [3, 5]. It is widely debated whether pyloric gland metaplasia is specific to Crohn’s disease as it is rarely observed in patients with ulcerative colitis with or without “backwash” ileitis [6-7]. Pyloric gland metaplasia can be a clinical marker for Crohn’s disease, similar to the detection of paneth cell metaplasia which occurs more frequently in ulcerative colitis [8].

## Conclusions

We report a patient with pyloric gland metaplasia in the setting of chronically active Crohn’s disease. To our knowledge, this is the first case to report pyloric metaplasia occurring at the site of the ileocecal valve. While pyloric gland metaplasia is not a specific marker, it continues to be a sensitive indicator of chronically active Crohn’s disease and a sign of ongoing inflammation. Clinicians should be aware of the significance of pyloric gland metaplasia as a factor in clinical and therapeutic decisions.
